# Alterations of Gut Microbiota in Cholestatic Infants and Their Correlation With Hepatic Function

**DOI:** 10.3389/fmicb.2018.02682

**Published:** 2018-11-13

**Authors:** Cheng Guo, Yinhu Li, Peipei Wang, Yingchao Li, Chuangzhao Qiu, Muxia Li, Daxi Wang, Ruiqin Zhao, Dongfang Li, Ye Wang, Shuaicheng Li, Wenkui Dai, Lin Zhang

**Affiliations:** ^1^Department of Pediatrics, The Third Hospital of Hebei Medical University, Shijiazhuang, China; ^2^Department of Computer Science, City University of Hong Kong, Kowloon Tong, Hong Kong; ^3^Department of Pediatrics, The Second Hospital of Hebei Medical University, Shijiazhuang, China; ^4^Department of Microbial Research, WeHealthGene Institute, Shenzhen, China; ^5^Department of Pediatrics, Children’s Hospital of Hebei Province, Shijiazhuang, China

**Keywords:** infantile cholestasis, 16S rRNA, hepatic function, bacterial biomarkers, co-abundance network

## Abstract

Cholestasis is a major hepatic disease in infants, with increasing morbidity in recent years. Accumulating evidence has revealed that the gut microbiota (GM) is associated with liver diseases, such as non-alcoholic steatohepatitis, cirrhosis, and hepatocellular carcinoma. However, GM alterations in cholestatic infants and the correlation between the GM and hepatic functions remain uninvestigated. In this study, 43 cholestatic infants (IC group) and 37 healthy infants (H group) were enrolled to detect GM discrepancies using 16S rDNA analysis. The diversity in the bacterial community was significantly lower in the IC group than that in the H group (*P* = 0.013). After determining the top 10 abundant genera of microbes in the IC and H groups, we found that 13 of them were differentially enriched, including *Bifidobacterium, Bacteroides, Streptococcus, Enterococcus*, and *Staphylococcus*. As compared with the H group, the IC group had a more complex GM co-occurrence network featured by three core nodes: *Phyllobacterium, Ruminococcus*, and *Anaerostipes*. In addition, the positive correlation between *Faecalibacterium* and *Erysipelatoclostridium* (*r* = 0.689, *P* = 0.000, FDR = 0.009) was not observed in the IC patients. Using the GM composition, the cholestatic patients can be distinguished from healthy infants with high accuracy [areas under receiver operating curve (AUC) > 0.97], wherein *Rothia, Eggerthella, Phyllobacterium*, and *Blautia* are identified as valuable biomarkers. Using KEGG annotation, we identified 32 functional categories with significant difference in enrichment of the GM of IC patients, including IC-enriched functional categories that were related to lipid metabolism, biodegradation and metabolism of xenobiotics, and various diseases. In contrast, the number of functions associated with amino acid metabolism, nucleotide metabolism, and vitamins metabolism was reduced in the IC patients. We also identified significant correlation between GM composition and indicators of hepatic function. *Megasphaera* positively correlated with total bilirubin (*r* = 0.455, *P* = 0.002) and direct bilirubin (*r* = 0.441, *P* = 0.003), whereas γ-glutamyl transpeptidase was positively associated with *Parasutterella* (*r* = 0.466, *P* = 0.002) and negatively related to *Streptococcus* (*r* = -0.450, *P* = 0.003). This study describes the GM characteristics in the cholestatic infants, illustrates the association between the GM components and the hepatic function, and provides a solid theoretical basis for GM intervention for the treatment of infantile cholestasis.

## Introduction

Cholestasis is a major hepatic disease in infants, with increasing incidence, with nearly one in every 2,500 individuals being affected (Fischler and Lamireau, 2014). Numerous studies have reported that infantile cholestasis (IC) can be caused by infection of the liver (e.g., hepatitis A, B, and C viral infection, Epstein-Barr virus infection, and cytomegalovirus infection) (Delemos and Friedman, 2013; Fawaz et al., 2017), abnormal structure of the biliary tract (e.g., biliary atresia and choledochal cyst) (Hoerning et al., 2014; Fawaz et al., 2017), hereditary diseases (e.g., Alagille syndrome, progressive familial intrahepatic cholestasis, and Aagenaes syndrome) (Hartley et al., 2013; Fawaz et al., 2017), and metabolic disorders [e.g., abnormal amino acid metabolism (Reichardt and Woo, 1991), abnormal carbohydrate metabolism (Phaneuf et al., 1991), and abnormal lipid metabolism (Vance, 2006)]. This disease can further injure hepatocytes, leading to hyperbilirubinemia (Brumbaugh and Mack, 2012), cirrhosis (Li et al., 2017) and may be fatal. As a key feature of IC, bile acids (BAs) closely interact with gut microbiota (GM) through the gut-liver axis (Li et al., 2017; Tripathi et al., 2018).

Previous research indicates that the GM participates in BA enterohepatic circulation and affects the secretion of BAs (Long et al., 2017; Tripathi et al., 2018). Bile salt hydrolases (BSH) are enzymes derived from the GM, which metabolize primary BAs into secondary BAs that, in turn, activate the synthesis of primary BAs through farnesoid X-activated receptor (FXR) and G protein-coupled BA receptor 1 (TGR5) in enterocytes (Long et al., 2017; Schneider et al., 2018). BAs also affect GM composition by controlling the PH of the gut environment, repressing the growth of pathogens and maintaining the balance of the GM (Islam et al., 2011).

To date, the association between GM alteration and hepatic diseases, including alcoholic fatty liver disease (ALD) (Cassard and Ciocan, 2017), non-alcoholic fatty liver disease (NAFLD) (Li et al., 2018), cirrhosis (Chen et al., 2011), and hepatocellular carcinoma (HCC) (Garrett, 2015), has been mainly studied in adults, whereas reports of studies in infants with immature GM are rare (de Muinck and Trosvik, 2018). Currently, the characteristics of GM in infants with IC and their association with hepatic function remain uninvestigated.

In this study, we enrolled a total of 43 IC patients and 37 healthy infants to investigate the roles of GM in the IC patients. In addition, to characterize the GM of the patients, we aimed to: (I) evaluate bacterial correlation and their contribution to hepatic function; (II) identify GM biomarkers for non-invasive diagnosis of IC; (III) elucidate GM discrepancy among patients with IC owing to different causes. These findings enhance our understanding of the pathogenic mechanism of dysbiotic GM, and provide a solid theoretical basis for GM intervention for the treatment of IC.

## Materials and Methods

### Ethics Statement

This study was approved by the Ethics Committee of The Third Hospital of Hebei Medical University under the registration number 2017-009-1. All the infants’ parents provided written informed consent, and volunteered to allow their children to participate in the investigation for scientific research.

### Participant Enrollment

The IC infants in this study were enrolled from the Third Hospital of Hebei Medical University, Children’s Hospital of Hebei Province, and Second Hospital of Hebei Medical University if they satisfied the following criteria: (I) age below 3 years; (II) levels of γ-glutamyl transpeptidase (GGT) were higher than 40 U/L, or the levels of total bilirubin (TBIL) were higher than 20 μmol/L (Cholestasis, 2015). In addition, the patients who met the following criteria were excluded from the study: (I) If their mother suffered from diabetes, high blood pressure, or chronic liver disease during the pregnancy; (II) If their mother had been continually exposed to drugs or probiotics during pregnancy or lactation; (III) If the patients suffered from allergic diseases (e.g., food allergy, eczema, and allergic gastroenteritis); (IV) If the patients had been exposed to antibiotic, probiotic, or proton pump inhibitors 4 weeks before fecal sample collection.

Healthy infants were selected from among the subjects if they passed infantile physical examinations of Third Hospital of Hebei Medical University and met the following standards: (I) The candidate should be younger than 3 years old; (II) The candidate should not have a history of allergic diseases (e.g., food allergy, eczema, and allergic gastroenteritis); (III) The candidate should not have had diarrhea 2 weeks prior to the study; (IV) The candidate should not have been administered any antibiotic, probiotic, or proton pump inhibitors 4 weeks prior to the study. Finally, 37 healthy infants (H group) and 43 cholestatic infants (IC group) were enrolled for the study between December 2016 and January 2018 (Table 1).

**Table 1:** Background information of participants.

	Healthy (*n* = 37)	Infantile cholestasis (*n* = 43)	*P*-value
**Gender**			0.967
Male	23	28	
Female	14	15	
**Age (mo)**	12.03 ± 8.75	2.25 ± 1.79	0.000
**Feeding pattern**			0.041
Breastfeeding	25	31	
Formula feeding	2	8	
Mixed	10	4	
**Delivery pattern**			0.011
Natural delivery	31	29	
Cesarean section	6	23	
**Indicators of hepatic function**			
TBA (1–10 μmol/L)^∗^	5.76 ± 2.43	146.71 ± 217.20	0.000
TBIL (3–20 μmol/L)^∗^	8.39 ± 4.38	133.60 ± 86.05	0.000
DBIL (2–6 μmol/L)^∗^	3.08 ± 1.16	90.46 ± 56.12	0.000
TP (60–80 g/L)^∗^	65.70 ± 4.78	56.01 ± 8.33	0.000
CHOL (0–5.2 mmol/L)^∗^	4.31 ± 0.46	3.43 ± 0.96	0.000
ALT (5–40 U/L)^∗^	14.30 ± 3.81	284.22 ± 354.20	0.000
AST (5–35 U/L)^∗^	24.27 ± 4.13	357.73 ± 467.64	0.000
GGT (7–40 U/L)^∗^	16.89 ± 4.43	212.39 ± 180.72	0.000


*^∗^TBA, total bile acids; TBIL, total bilirubin; DBIL, direct bilirubin; TP, total protein; CHOL, total cholesterol; ALT, serum alanine aminotransferase; AST, aspartate aminotransferase; GGT, γ-glutamyl transpeptidase.*

### Sample Collection

Fresh stools from the IC patients were collected in the morning after their admission to the hospital, and fresh stools from the healthy subjects were collected during their physical examination. The blood samples were collected from the participants, and hepatic function was examined using the blood autoanalyzer (Beckman Coulter AU5800, Brea, CA, United States). The clinical indices for the assessment of hepatic function consist of total bile acids (TBA), total bilirubin (TBIL), direct bilirubin (DBIL), total protein (TP), total cholesterol (CHOL), serum alanine aminotransferase (ALT), aspartate aminotransferase (AST), and γ-glutamyl transpeptidase (GGT) (Supplementary Table S1).

### DNA Extraction, Library Construction, and Sequencing

Bacterial DNA was extracted from stools using the E.Z.N.A.^®^ Soil DNA Kit (Omega BioTek, Norcross, GA, United States) according to the manufacturer’s protocols. The V3–V4 region of the 16S rRNA gene was amplified by primers 338F and 806R, using the PCR kit (TransGenAP221-02, Peking). The quality of the PCR product was determined (Qubit, Thermo Fisher Scientific, Singapore), and it was then prepared for library construction (TruSeq DNA PCR-Free kit, Illumina, San Diego, CA, United States). Then the eligible libraries were paired-end sequenced as 300 (nt) reads using the MiSeq platform (Illumina, San Diego, CA, United States). The raw reads were uploaded to the NCBI Sequence Read Archive (SRA) Database (Accession Number: SRP151718).

### Taxonomical Annotation

Raw reads were filtered if they contained more than 10 low-quality (<Q20) bases, or a 15-base adapter contamination owing to a self-edited program. The paired reads were connected into tags on the basis of an overlap of at least 50 bases. Then, the tags were clustered into Operational Taxonomic Units (OTUs) with 97% similarity using the USEARCH (v7.0.1090) program. After the elimination of the chimeras, the OTUs were aligned to the RDP 16S rRNA databases (trainset 16/release 11.5) (Cole et al., 2014) and their corresponding taxonomic positions were defined. The Shannon index was calculated using the “vegan” package in R (version 3.4.1).

### PERMANOVA Analysis

The impact of physical indices (e.g., gender, age, delivery pattern, and feeding pattern) on GM distributions was assessed using Permutational Multivariate Analysis of Variance (PERMANOVA) (Tang et al., 2016) with 9,999 permutations and Euclidean distances (package “vegan” in R).

### Selection of Biomarkers and Validation Test

For biomarker identification, a two-step schema was adopted. First, a random forest model (Liaw and Wiener, 2002) was constructed for the discrimination between the H and IC groups, and candidate biomarkers were selected on the basis of the Gini values and optimal variation numbers (using the R package “random-Forest”). Second, the GM between the two groups was compared using the Wilcoxon rank-sum test (using “wilcox.test” in R). Candidate biomarkers with a significant adjusted difference (*P* < 0.05, FDR < 0.05) were selected as final biomarkers for cholestatic patients.

All the samples were randomized into two sets (one training set and one testing set). The training set was used to construct a random forest model and the test set was used to validate final biomarkers. The accuracy of biomarkers for screening of cholestatic infants was estimated using area under the curve (AUC) values with five repeats (using the R package “pROC”).

### Functional Prediction and Enrichment

Gut microbiota functions were predicted on the basis of 16S rRNA OTUs profiling using PICRUST with default setting (Langille et al., 2013). KEGG Orthology (KO) abundances were calculated for each sample, and the abundances of functional categories on level III of the KEGG database were detected. The differentially enriched categories between the H group and the IC group were identified using the Wilcoxon rank-sum test (*P* < 0.05). The associations between the KEGG pathways and the clinical indices were estimated with Spearman coefficient using “cor” in R.

### Statistical Analysis

The Wilcoxon rank-sum test (using “wilcox.test” in R) was used to detect differentially enriched genera between the H and IC groups (*P* < 0.05). The Spearman correlation analysis was executed on the genus level, and the relationships whose *r*-values were higher than 0.6 or lower than -0.6 were retained. The co-occurrence networks were visualized using the Cytoscape software (v2.2.0) (Shannon et al., 2003). For the 43 IC patients and the 37 healthy infants, the relationships between the GM and eight clinical indices were evaluated using the Spearman coefficient. The statistical results from the Wilcoxon rank-sum test and Spearman correlation analysis were adjusted with the Benjamini and Hochberg method (FDR < 0.05) using “p.adjust” in R.

## Results

### Sample Characteristics and Data Output

**FIGURE 1:**
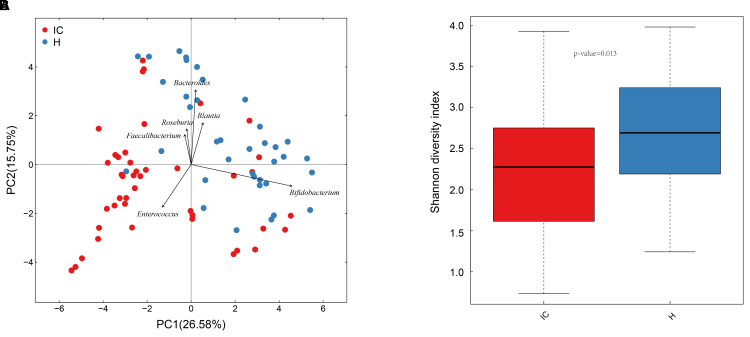
Principal component analysis (PCA) distribution and bacterial diversity in IC and healthy infants. **(A)** Using PCA analysis, the samples from the IC group were clustered together, and they were separated from those of the H group. The GM of the participants predominantly included *Bacteroides, Bifidobacterium*, and *Enterococcus*. **(B)** The diversity in the bacterial community was significantly lower in the IC infants (2.222 ± 0.790) than that in the H group (2.669 ± 0.753) (*P* = 0.013).

A total of 37 healthy infants (H group) and 43 cholestatic infants (IC group) were enrolled for stool sample collection (Table 1). Among the IC patients, 5 had cholestasis due to cytomegalovirus hepatitis, 9 had cholestasis due to biliary atresia, and the rest of the patients were diagnosed without any discernible cause (Supplementary Table S1). 16S rRNA sequencing of the samples and the connection of high-quality pair-end reads finally yielded 17,889 ± 5,012 (mean ± SD) tags, which ranged from 4,863 to 25,213. The number of OTUs ranged from 112 to 244 for the H group and from 54 to 245 for the IC group. After RDP database alignment, 93 genera of 7 phyla were identified from the samples, and the feeding pattern of the infants had an impact on the difference in GM between the H and IC groups (*P* = 0.009, PERMANOVA analysis, Supplementary Table S2). All the samples predominantly showed the following genera: *Bifidobacterium, Bacteroides, Enterococcus, Blautia, Roseburia*, and *Faecalibacterium* (Figure 1A). Principal component analysis (PCA) showed that the samples from the IC group clustered together and were separated from the H group (Figure 1A). Moreover, the IC patients exhibited a significantly lower diversity in the bacterial community: the average value of the Shannon index was 2.222 ± 0.790 for the IC group and 2.669 ± 0.753 for the H group (*P* = 0.013, Figure 1B).

### IC and H Groups Showed Discrepancy in GM Structure, and GM Biomarkers Were Identified for Screening of IC Infants

**FIGURE 2:**
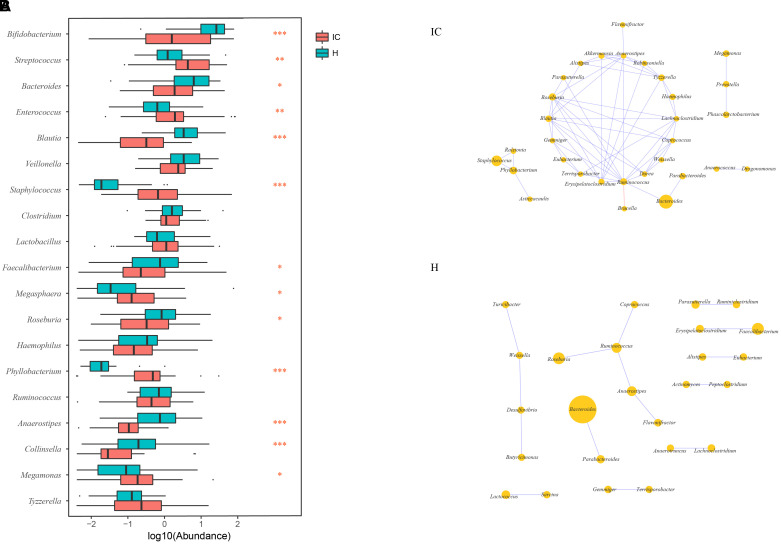
Discrepancy in GM components and networks between the IC and H groups. **(A)** A total of 13 taxa are differentially enriched between the IC and H groups. *Streptococcus, Enterococcus, Staphylococcus, Megasphaera, Phyllobacterium*, and *Megamonas* are enriched in the IC infants, whereas the numbers of *Bifidobacterium, Bacteroides, Blautia, Faecalibacterium, Roseburia, Anaerostipes*, and *Collinsella* are lower than those in the H group. One, two, and three asterisks stand for the *P*-value lesser than 0.05, 0.01, and 0.001, respectively. **(B)** The GM co-occurrence network was constructed for the IC and H groups, respectively. The purple and red edges stand for the positive and negative correlations, respectively. The diameter of the circle is proportional to the relative abundance. The bacterial network in the IC group is more complicated than that in the H group, and some correlations in the H group are disrupted.

Among the top 10 abundant genera found in the IC and H groups, 13 were differentially enriched (Figure 2A). *Streptococcus* (10.449 ± 13.479%, *P* = 0.002, FDR = 0.002), *Enterococcus* (8.301 ± 20.546%, *P* = 0.003, FDR = 0.003), *Staphylococcus* (3.520 ± 11.728%, *P* = 0.000, FDR = 0.000), *Megasphaera* (0.443 ± 0.755%, *P* = 0.018, FDR = 0.018), *Phyllobacterium* (1.401 ± 4.770%, *P* = 0.000, FDR = 0.000), and *Megamonas* (0.841 ± 3.248%, *P* = 0.0124, FDR = 0.013) were found to be enriched in the IC infants. Conversely, the relative abundance of *Bifidobacterium* (14.006 ± 21.753%, *P* = 0.000, FDR = 0.000), *Bacteroides* (5.699 ± 9.514%, *P* = 0.026, FDR = 0.026), *Blautia* (0.788 ± 1.313%, *P* = 0.000, FDR = 0.000), *Faecalibacterium* (2.482 ± 8.204%, *P* = 0.041, FDR = 0.041), *Roseburia* (1.215 ± 2.270%, *P* = 0.017, FDR = 0.018), *Anaerostipes* (0.143 ± 0.296%, *P* = 0.000, FDR = 0.000), and *Collinsella* (0.351 ± 1.412%, *P* = 0.000, FDR = 0.000) was reduced in the IC group (Figure 2A).

The GM co-occurrence networks were constructed for the H and IC groups, respectively, and the cholestatic infants showed a greater complexity in networks (Figure 2B). For healthy infants, *Ruminococcus* was the core node of the network, and enrichment of *Faecalibacterium* was positively associated with *Erysipelatoclostridium* (*r* = 0.689, *P* = 0.000, FDR = 0.009). Such a correlation was not observed in the IC group. Instead, the IC group contained three novel positive correlations between *Bacteroides* and *Ruminococcus* (*r* = 0.611, *P* = 0.000, FDR = 0.048), *Staphylococcus* and *Phyllobacterium* (*r* = 0.672, *P* = 0.000, FDR = 0.048), *Megamonas*, and *Prevotella* (*r* = 0.638, *P* = 0.000, FDR = 0.015) (Figure 2B). *Phyllobacterium, Ruminococcus*, and *Anaerostipes* were the core nodes of co-occurrence in the network for the IC group.

**FIGURE 3:**
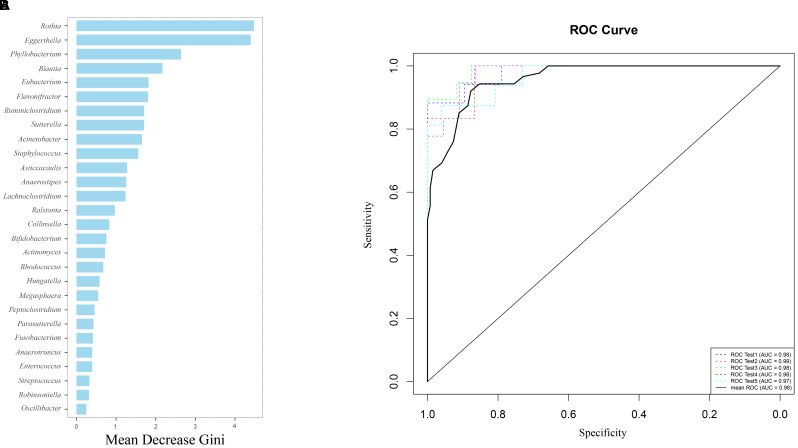
Gut microbiota (GM) biomarkers help to differentiate between the IC and H groups. **(A)** Following optimal variation numbers were indicated by random forest classifiers, 28 GM biomarkers were examined for the IC group as compared with those for the H group. Their Gini values are shown in the picture. **(B)** The accuracy of biomarkers was verified using cross-validation, and their AUC values were calculated. The ROC curves were drawn with five repeats by different colors.

Using the Random forest classifier, 28 biomarkers were identified to differentiate IC infants from healthy infants (Figure 3A) with high accuracy (AUC > 0.97, Figure 3B). Among them, *Rothia* (Gini = 4.480), *Eggerthella* (Gini = 4.399), *Phyllobacterium* (Gini = 2.637), and *Blautia* (Gini = 2.172) were found to be the four genera with the highest Gini values, and helped in distinguishing between the two groups.

### GM Functional Categories Were Differentially Enriched Between the H and IC Groups

**FIGURE 4:**
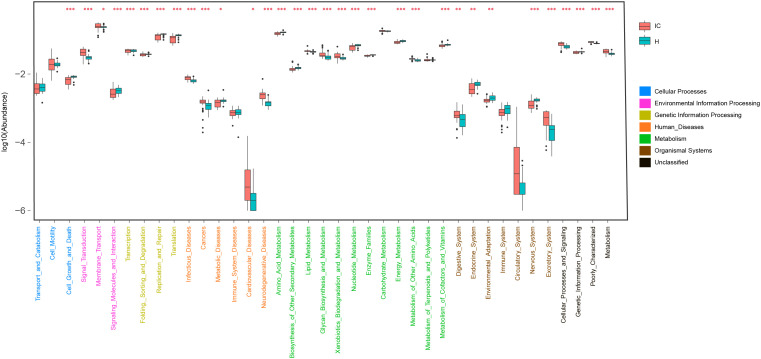
Distribution of KEGG level II pathways in the IC and H groups. Relying on the functional classifications of the KEGG database, the enriched pathways were determined for the IC and H groups, and the functional categories between these two groups were compared. *P*-values are indicated by asterisks on the top (one, two, and three asterisks stand for a *P*-value smaller than 0.05, 0.01, and 0.001, respectively). In addition, level I classification of these KEGG functional categories is suggested by different colors on the right.

Using KO distributions of GM in all the infants, 32 differentially enriched KEGG functional modules between the IC and H groups were identified (Figure 4). The enriched functional categories in the IC patients included “Lipid metabolism” (*P* = 0.000, FDR = 0.000), “Glycan biosynthesis and metabolism”(*P* = 0.000, FDR = 0.000), and “Xenobiotics biodegradation and metabolism” (*P* = 0.000, FDR = 0.000) (Figure 4). Contrastingly, the abundance of “Amino acid metabolism” (*P* = 0.000, FDR = 0.000), “Nucleotide metabolism” (*P* = 0.000, FDR = 0.000), and “Metabolism of cofactors and vitamins” (*P* = 0.000, FDR = 0.000) was reduced, and more bacterial genes participated in the hosts “Digestive system” (*P* = 0.000, FDR = 0.000) and “Excretory system” (*P* = 0.000, FDR = 0.000) in the IC group (Figure 4). We also found that the IC-enriched functional modules were associated with the occurrence of diseases, including infectious diseases (*P* = 0.000, FDR = 0.000), metabolic diseases (*P* = 0.000, FDR = 0.000), cardiovascular diseases (*P* = 0.000, FDR = 0.000), neurodegenerative diseases (*P* = 0.000, FDR = 0.000), and even cancer (*P* = 0.000, FDR = 0.000). Additionally, the GM of the IC group showed enrichment in “Signal transduction” (*P* = 0.000, FDR = 0.000), “membrane transport” (*P* = 0.000, FDR = 0.000), and “Cellular processing and signaling” (*P* = 0.000, FDR = 0.000) (Figure 4).

### Taxonomic and Functional Composition of the GM Were Correlated With Hepatic Function

**FIGURE 5:**
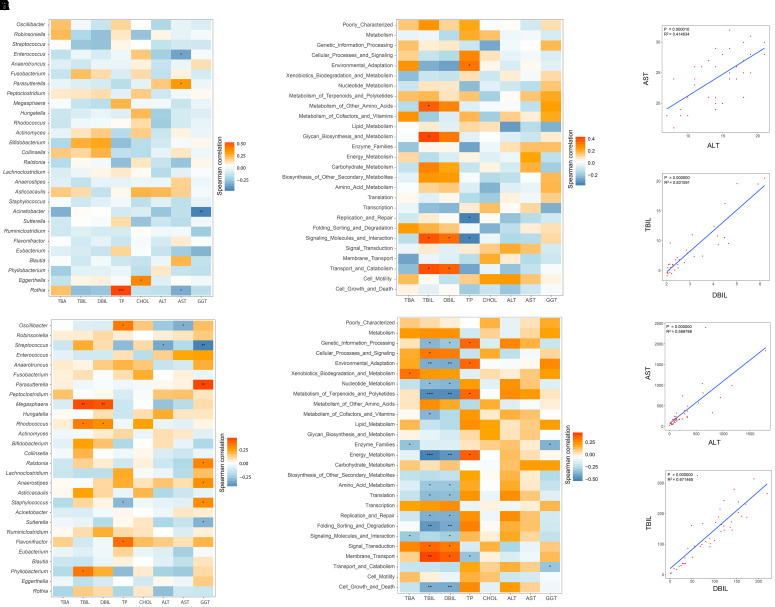
Relationships between GM and hepatic function, and associations among indicators of hepatic function. Spearman correlation analysis was performed between GM components and eight indicators of hepatic function, and the results for the H and IC groups are shown in panels **(A,D)**, respectively. The relationships between the GM functional categories and the hepatic function are shown for healthy infants **(B)** and IC patients **(E)**. In these pictures, orange and blue colors stand for the positive and negative relationships, respectively. One, two, and three asterisks stand for a *P*-value smaller than 0.05, 0.01, and 0.001, respectively. In panels **(C,F)**, the significant relationships (*r* > 0.5 or *r* < –0.6, *P* < 0.05) among different indicators of hepatic function are shown for the H and IC groups.

Pairwise correlations between GM genera and eight clinical indicators of hepatic function were estimated in healthy infants (Figure 5A). AST negatively and positively correlated with *Enterococcus* (*r* = -0.356, *P* = 0.031) and *Parasutterella* (*r* = -0.330, *P* = 0.046). Negative associations between GGT and *Acinetobacter* (*r* = -0.458, *P* = 0.004), AST and *Rothia* (*r* = -0.332, *P* = 0.045) were also found. In contrast, the relationships between the GM components and the hepatic functional indices were also constructed for the 43 cholestatic infants, and altered associations were observed (Figure 5D). For instance, *Oscillibacter* negatively correlated with AST (*r* = -0.322, *P* = 0.0350), whereas it was positively associated with TP (*r* = 0.352, *P* = 0.021). *Streptococcus* was also negatively associated with ALT (*r* = -0.342, *P* = 0.025) and GGT (*r* = -0.450, *P* = 0.0026). Positive correlations were found between *Megasphaera* and indicators of hepatic synthesis [TBIL (*r* = 0.455, *P* = 0.002), and DBIL (*r* = 0.441, *P* = 0.003)]. In addition, *Parasutterella* positively correlated with GGT (*r* = 0.466, *P* = 0.002).

The associations between the GM functional categories and the hepatic function were also investigated (Figures 5B,E). For healthy infants, TBIL positively correlated with “Metabolism of other amino acids” (*r* = 0.373, *P* = 0.023) and “Glycan biosynthesis and metabolism” (*r* = 0.413, *P* = 0.011) (Figure 5B). TP was negatively associated with the bacterial function “Replication and repair” (*r* = -0.351, *P* = 0.034), and “Signaling molecules and interaction” (*r* = -0.349, *P* = 0.035). In the IC group, the functional items “Metabolism of terpenoids and polyketides” and “Energy metabolism” negatively correlated with TBIL (*r* = -0.489 and *r* = -0.515, respectively) and DBIL (*r* = -0.486 and *r* = -0.458, respectively), and both of them positively correlated with TP (*r* = 0.350 and *r* = 0.370, respectively) (Figure 5E). Positive associations were identified between “Membrane transport” and indicators of hepatic synthesis [TBIL (*r* = 0.372, *P* = 0.005) and DBIL (*r* = 0.372, *P* = 0.014)]. In addition, AST positively correlated with ALT (*r* = 0.590, *P* = 0.000), and TBIL was positively associated with DBIL (*r* = 0.672, *P* = 0.000) (Figures 5C,F).

### No Apparent GM Difference Was Detected Between IC Cohorts With Different Causes

The IC patients were sorted on the basis of clinical causes for their condition: biliary atresia (BA-IC cohort) or cytomegalovirus hepatitis (CMV-IC cohort). These two cohorts exhibited no significant difference in bacterial diversity (2.259 ± 0.912 and 1.956 ± 0.962 for the BA-IC and CMV-IC cohorts, respectively, Supplementary Figure S1A). We did not detect any significant differences in GM composition (Supplementary Figure S2B).

**FIGURE 6:**
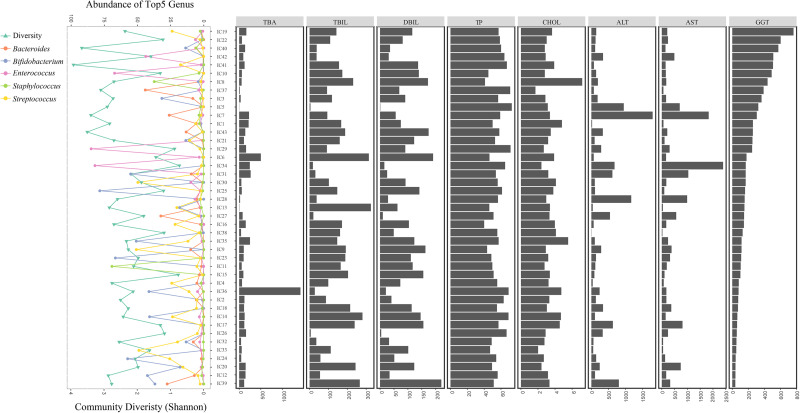
Distribution of GM components and hepatic function in all IC patients. All IC patients were sorted into groups based on decreasing GGT concentrations. The distribution of bacterial diversity and top 5 genera in each sample is shown on the left. Levels of eight hepatic indicators are indicated by the histograms on the right.

With decreasing GGT levels, the distribution of the diversity in the bacterial community, top 5 genera, and hepatic functional indicators were determined for the 43 IC patients (Figure 6). Special incidence was found although the GM-liver associations were illustrated. With low abundance of *Bifidobacterium* (0.391%) and *Bacteroides* (0.181%), the levels of GGT (324U/L) were found to be high in IC5; however, the concentrations of TBA (3.2 μmol/L) remained normal (Figure 6).

## Discussion

In this study, we mainly elucidated the discrepancy in GM between the IC patients and the healthy infants. Between the two groups, we observed lower diversity in the bacterial community in the IC patients, which is probably associated with the reduced inflow of BAs (Fischler and Lamireau, 2014) and GM dysbiosis (Islam et al., 2011). Although significant differences in age, delivery pattern, and feeding pattern were detected between the IC and H groups (*P* < 0.05), inter-group GM discrepancy was mainly attributed to the feeding pattern after PERMANOVA analysis (Supplementary Table S2), and the results also emphasized the impact of diet on GM components (Sonnenburg et al., 2016).

Analysis of GM composition showed low proportions of *Bifidobacterium, Bacteroides*, and *Faecalibacterium* in IC patients. Long et al. (2017) study reported that *Bifidobacterium* and *Bacteroides* could secrete BSH enzymes, which liberate conjugated bile acids and facilitate BA enterohepatic circulation. Furthermore, *Bifidobacterium* (Odenwald and Turner, 2017) and *Faecalibacterium* (Miquel et al., 2013) could reinforce the barrier integrity of epidermal cells (Odenwald and Turner, 2017), and repress systemic inflammation reactions through the production of SCFAs (Li et al., 2017). The low abundance of *Bifidobacterium* and *Bacteroides* partly explained the high GGT levels and severe liver injury in IC5. In addition, increased *Streptococcus* numbers in IC patients might raise the levels of TNF-α, IL-6, and IFN-γ, and hence, contribute to systematic inflammations (Jiang et al., 2015). Similarly, in adult patients with cholestatic liver disease, the IC patients showed an increased number of *Enterococcus*; however, overrepresented *Lactobacillus* and *Fusobacterium* were not found in the infantile patients (Sabino et al., 2016), which also suggested a different dysbiotic pattern in infants and adults with related diseases.

Differentiated GM composition in IC patients also contributes to its unique co-occurrence network. For instance, a novel positive association between *Ruminococcus* and *Bacteroides* was discovered in the IC patients. As *Ruminococcus* can generate ursodeoxycholic acid (UDCA) to remiss cholestasis (Lee et al., 2013), decreased *Bacteroides* in IC patients suggest a reduction in *Ruminococcus* numbers, which might further aggravate cholestasis. Another core node for IC patients, *Anaerostipes* was found to contribute to host health improvement by producing SCFAs (Strati et al., 2016), which warranted further investigation in its potential associations with IC. These results suggest that the dynamic changes in GM co-occurrence networks in IC infants correspond to their health status.

Based on GM discrepancy, 28 GM biomarkers were identified for the diagnosis of IC with high precision. As the major biomarkers, *Eggerthella* can produce ω muricholic acid (ωMCA), which can be processed for deoxycholic acid (DCA) synthesis (Long et al., 2017), and participates in inflammation and insulin signaling (Wahlstrom et al., 2016). Therefore, GM biomarkers provided a promising approach for non-invasive diagnosis of IC.

A functional comparison between the H and IC groups revealed differential nutrient metabolism in the GM of IC patients. In the IC patients, more undigested lipids accumulate in the large intestine upon reduction in levels of BAs (Fischler and Lamireau, 2014), and become an important energy source for GM. This hypothesis explained the increase in relative abundance of lipid metabolic modules in IC patients. In addition, elevated levels of functional categories in biodegradation of xenobiotics might be related to the overgrowth of pathogens (Islam et al., 2011) due to the decrease in influx of BAs (Fischler and Lamireau, 2014). Previous research suggests that gut pathogens can be inhibited by BAs through spore germination (Sorg and Sonenshein, 2010) and by regulating vegetative cells (Buffie et al., 2015). With a reduction in the influx of BAs, the increase in number of pathogens might promote toxin secretion, aggravate GM dysbiosis, and injure the immune system (Abt et al., 2016), which also explained the increasing risks of infectious diseases, metabolic diseases, cardiovascular diseases, and neurodegenerative diseases in IC patients.

Discrepancies in relationships between GM and indicators of hepatic functions were further seen in IC infants. *Megasphaera* disrupts metabolic functions of the liver (suggested by TBIL and DBIL), via an unknown mechanism (Lv et al., 2016), which supports their positive correlation in IC patients. Correlating positively with inflammatory cells, *Parasutterella* triggers inflammatory responses (Chen et al., 2018), and positively correlates with indicators of hepatic injury (suggested by GGT). In healthy infants, such relationships were not found as the GM component was shaped by other factors than BAs, such as diet (Sonnenburg et al., 2016), delivery pattern (Rutayisire et al., 2016), and ethnicity (Gupta et al., 2017). Without apparent GM differences among IC patients with different causes for their condition, we suspect that similar GM alterations are driven by the reduced influx of BAs (Fischler and Lamireau, 2014). GM intervention can be adopted to ameliorate hepatic burdens and IC symptoms through the liver-BAs-microbiota associations.

This study presents data for GM alterations in IC patients, provides GM biomarkers for IC diagnosis, and describes the associations between bacterial commensals and hepatic function. However, there are some limitations to the research: (I) A greater number of IC patients with different causes for their condition should be enrolled; (II) GM biomarkers should be validated in populations of different ethnicities. In further studies, additional work is required, such as: (I) A large-cohort study needs to be performed to test the identified biomarkers; (II) Alterations in the microbiome and metabolite alterations in the GM of IC patients need to be investigated; (III) Changes in the immune system and its correlation with GM need be determined. In summary, this research provides a better understanding of the pathogenesis of IC, and emphasizes the therapeutic potential of GM in IC intervention.

## Author Contributions

LZ and WD managed the project. CG, PW, YCL, and RZ performed the sampling and information collection. ML and YW prepared the DNA. YHL, CQ, and DL performed the bioinformatics analysis in this work. CG and YHL interpreted the analysis results and wrote the paper. SL, DW, and WD optimized the graphs. SL, WD, DL, and LZ guided the statistic analysis and polished the article. All authors reviewed the manuscript.

## Conflict of Interest Statement

The authors declare that the research was conducted in the absence of any commercial or financial relationships that could be construed as a potential conflict of interest.

## Supplementary Material

FIGURE S1 Comparison on bacterial diversity and GM components between IC cohorts with different causes. **(A)** No significant difference in bacterial diversity was detected between BA-IC cohort and CMV-IC cohort. **(B)** No taxa was differentially enriched between BA-IC and CMV-IC cohorts.

TABLE S1 Detailed background information of the participants.

TABLE S2 PERMANOVA ranking associations between GM and indicators of hepatic function.
