# Corrigendum: Alterations of Gut Microbiota in Cholestatic Infants and Their Correlation With Hepatic Function

**DOI:** 10.3389/fmicb.2020.01599

**Published:** 2020-07-24

**Authors:** Cheng Guo, Yinhu Li, Peipei Wang, Yingchao Li, Chuangzhao Qiu, Muxia Li, Daxi Wang, Ruiqin Zhao, Dongfang Li, Ye Wang, Shuaicheng Li, Wenkui Dai, Lin Zhang

**Affiliations:** ^1^Department of Pediatrics, The Third Hospital of Hebei Medical University, Shijiazhuang, China; ^2^Department of Computer Science, City University of Hong Kong, Kowloon Tong, Hong Kong; ^3^Department of Pediatrics, The Second Hospital of Hebei Medical University, Shijiazhuang, China; ^4^Department of Microbial Research, WeHealthGene Institute, Shenzhen, China; ^5^Department of Pediatrics, Children's Hospital of Hebei Province, Shijiazhuang, China

**Keywords:** infantile cholestasis, 16S rRNA, hepatic function, bacterial biomarkers, co-abundance network

In the original article, there was an error in the Funding statement. The correct name for the funder is **Natural Science Foundation of Hebei Province** (No. H2018206310).

The authors apologize for this error and state that this does not change the scientific conclusions of the article in any way. The original article has been updated.

