# Supplementary figures and images for: Alterations of Gut Microbiota in Cholestatic Infants and Their Correlation With Hepatic Function
Source: Front Microbiol. 2018 Nov 13;9:2682. doi: 10.3389/fmicb.2018.02682 (PMC6243132; doi:10.3389/fmicb.2018.02682)

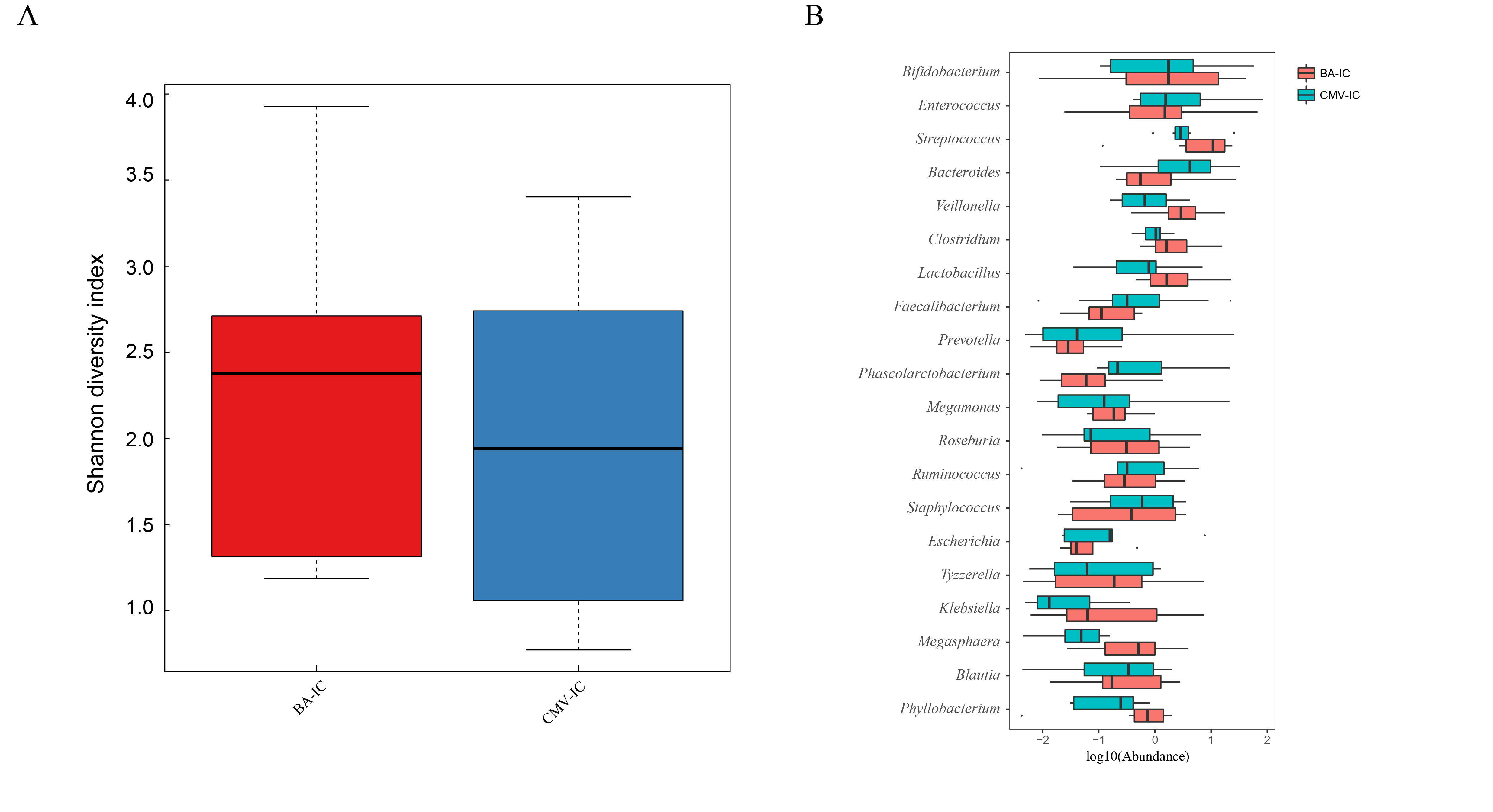

Supplement: FIGURE S1 — Comparison on bacterial diversity and GM components between IC cohorts with different causes. (A) No significant difference in bacterial diversity was detected between BA-IC cohort and CMV-IC cohort. (B) No taxa was differentially enriched between BA-IC and CMV-IC cohorts. [file Image_1.TIF]
